# A comparative study of the radial pulse between primary dysmenorrhea patients and healthy subjects during the menstrual phase

**DOI:** 10.1038/s41598-019-46066-2

**Published:** 2019-07-04

**Authors:** Jihye Kim, Jang-Han Bae, Boncho Ku, Mi Hong Yim, Lin Ang, Hyunho Kim, Young Ju Jeon

**Affiliations:** 1 Clinical Research Coordinating Team, Korea Institute of Oriental Medicine, 1672 Yuseongdae-ro, Yuseong-gu, Daejeon Republic of Korea; 2 Future Medicine Division, Korea Institute of Oriental Medicine, 1672 Yuseongdae-ro, Yuseong-gu, Daejeon Republic of Korea; 3 Clinical Medicine Division, Korea Institute of Oriental Medicine, 1672 Yuseongdae-ro, Yuseong-gu, Daejeon Republic of Korea; 4 Korea Convergence Medicine, University of Science and Technology, 217 Gajeong-ro, Gajong-dong, Yuseong-gu, Daejeon Republic of Korea; 5 Dongshin Korean Medicine Hospital (Mokdong, Seoul), 351, Omok-ro, Yangcheon-gu, Seoul Republic of Korea

**Keywords:** Physical examination, Reproductive signs and symptoms

## Abstract

The aim of this study was to compare radial pulse characteristics between primary dysmenorrhea (PD) patients and healthy subjects throughout the menstrual cycle. A total of 48 females aged 20 to 29 years participated, and all subjects were assigned to two groups according to their visual analogue scale scores. The radial pulse of each subject was obtained using a pulse tonometric device during menstrual, follicular, and luteal phases. In addition, various pulse analysis indices were used to estimate the pulse characteristics. The pulse tension index (PTI) and pulse depth index (PDI) in the patient group were significantly lower than those in the healthy group during the menstrual phase (P < 0.01 and <0.001, respectively). According to univariate logistic regression results, the PTI, PDI and optimal applied pressure (OAP) were significantly correlated with PD, and the model based on the PTI and OAP performed best (AUC = 0.828). This study is the first to analyze pulse tension inferred from the PTI and to apply this parameter to clinical practice. The results of this study confirmed the possibility of quantitatively measuring pulse tension and suggest that the PTI and OAP can serve as potential clinical indicators for pain disorders.

## Introduction

Pulse diagnosis can be used to diagnose physiological conditions and predict the prognosis of a pathological disorder^1^. Traditional Korean medicine (TKM) and traditional Chinese medicine (TCM) physicians perform pulse diagnosis by adjusting the pressure applied by their fingertips at three parts of the radial artery—*cun*, *guan*, and *chi—*of the patient’s wrist^1^. TKM physicians diagnose and provide prescriptions to patients on the basis of pulse characteristics; however, serious problems can arise due to reliance on the physician’s subjective point of view. The accuracy of pulse diagnosis depends on the individual’s experience, observational skills, and fingertip sensitivity^2^. Moreover, there is no clear diagnostic criterion, and it is difficult to define physical variables that reflect pulse characteristics. Recently, to overcome these problems, several scientific advancements have been achieved to quantify pulse diagnostic parameters, including the development of a pulse tonometric device (PTD)^3^. Various clinical studies using a PTD have been conducted with various variables for specific disorders, such as hypertension^4^, coronary heart disease^5^, diabetes mellitus^6^, cirrhosis^7^, and pancreatitis^8^. However, few studies have used a PTD for gynecological diseases.

Dysmenorrhea, also known as menstrual cramps, is pain during menstruation and is related to a wide range of psychiatric symptoms, especially depression, anxiety, psychosocial stress and a poor quality of life^9^. Dysmenorrhea is also one of the clinical signs of endometriosis, which is a more intense disorder and corresponds to the severity of pain^10^. In addition, as pelvic and lower abdominal pain represents the key symptom of many gynecological diseases, it is important to have a correct diagnosis and appropriate treatment of primary dysmenorrhea due to the complex nature of the disease^11^. In young women, dysmenorrhea often occurs without an underlying problem, a condition known as primary dysmenorrhea (PD). In older women, dysmenorrhea is more often due to underlying issues such as uterine fibroids, adenomyosis, or endometriosis, a condition known as secondary dysmenorrhea^12^. PD is a common gynecological complication among women of reproductive age. Studies have shown that pain-related gynecological complications have a negative effect on psychological health, which may eventually lead to certain limitations in social activities and cause a significant impact on women’s mental health and quality of life^13^. Nonsteroidal anti-inflammatory drugs or oral contraceptive pills are widely used as first-line therapy for women with PD. However, these drugs may have side effects in some women and may not provide any long-term pain relief^14^. Therefore, many patients with PD are seeking alternative treatment through TKM and TCM, such as acupuncture, moxibustion, or herbal medicine^15^. Jeon *et al*.^16^ investigated whether radial pulse indices derived from a PTD were related to menstrual pain during menstrual and nonmenstrual periods. They reported that women with menstrual pain had decreased blood vessel elasticity, low pulsatile force and a reduced diastolic phase in the pulse wave during the nonmenstrual period^16^. Su *et al*.^17^ also reported that the amplitudes of the main wave and dicrotic wave and the systolic area of the radial pulse waveforms were greater in the luteal phase than those in the follicular phase. Chen *et al*.^18^ observed that pain could increase the width of the upper 1/3 of the main wave, the amplitude of the gorge of the main wave and the dicrotic notch but decrease the time of the gorge of the main wave and the amplitude of the dicrotic wave; therefore, they concluded that pulse waveform parameters change in opposite directions when PD patients experience pain and when they experience pain relief.

However, several limitations were identified in these prior works that used PTD in PD patients. First, pattern identification was not considered for the PD patients in these studies. In TKM, treatment methods for PD can be designed and tailored according to identified patterns, such as stagnation of *qi* and *blood*, coagulation of cold-dampness, deficiency of *blood* and *qi*, or impairment of the liver and kidneys^19,20^. TKM physicians identify patterns of PD according to clinical information acquired through inspection, listening, inquiring and palpation^21^. Among this clinical information, pulse characteristics are one of the most important elements. As pulse characteristics vary according to the pattern of the disease, pattern identification should be considered in the pulse diagnostic research in TKM for a higher diagnosis accuracy and better clinical application. Second, all phases of the menstrual cycle were not considered for the PD patients in previous studies. Jeon SH measured the radial pulse during the follicular and menstrual phases, whereas Su SY measured the radial pulse during the late follicular and late luteal phases^16,17^. Additionally, Chen WH measured the radial pulse during the luteal and menstrual phases^18^. However, no studies considered all three phases (menstrual, follicular and luteal phases) of the menstrual cycle. Such considerations are necessary because characteristics of the radial pulse change throughout the entire menstrual cycle^22^. Third, suitable analytical indices to investigate the pulse characteristics of PD patients have not been fully considered. In TKM and TCM, important pulse properties include the pulse depth, width, length, regularity, and tension^23^. Although many studies have been conducted to quantify these pulse properties, several indices have been unclearly calculated, which have yielded uncertain meanings, and the directly related variables were not used in prior fragmented studies^24^. In particular, an analytical index reflecting pulse tension characteristics has not been used, even though pulse tension is a major pulse characteristic of patients with pain. Therefore, a PD study using a PTD for this objective and suitable indicators is required. The aims of this study were to compare radial pulse characteristics between PD patients and healthy subjects throughout the menstrual cycle and to investigate the effects of menstrual pain on the radial pulse. To this end, a novel indicator reflecting the pulse characteristics of PD patients was considered.

## Results

### Comparison of baseline characteristics between the PD patient and healthy subject groups

Of the 48 subjects recruited, only the data sets of 47 subjects were analyzed. One participant was excluded from the data set because she violated the protocol of this study. The remaining subjects were grouped into the healthy group, which included 24 women, and the patient group, which included 23 women. The general characteristics of the participants in both groups are presented in Table 1. The mean ages of the patient and healthy subject groups were 23.5 ± 2.5 and 22.9 ± 1.6, respectively, with no statistically significant differences between the two groups. Additionally, no significant differences were found in anthropometric information, vital signs or substances used, including caffeine and alcohol, between the groups. Moreover, no statistically significant differences existed between the means of the two groups’ menstrual characteristics. The VAS score was used as an allocation criterion during the screening process for this study. A significant difference was observed in the maximum VAS score between the PD patients and healthy subjects. In addition, the MMP scores were significantly different between the PD patients and healthy subjects. The CMSS also indicated significant differences in the duration and severity of menstrual pain between the PD patients and healthy subjects. Furthermore, twenty-two patients were diagnosed with stagnation of *qi* and *blood*, whereas only one of the 23 patients was diagnosed with deficiency of *blood* and *qi*.

**Table 1:** Baseline characteristics of the study participants.

	Healthy subjects	PD patients	P-value
N	24 (51.1%)	23 (48.9%)	
Age (year)	22.9 ± 1.6	23.5 ± 2.5	0.324
**Anthropometric data**			
Height (cm)	160.6 ± 4.4	162.3 ± 4.0	0.161
Weight (kg)	52.9 ± 6.1	54.9 ± 4.9	0.223
Body mass index (kg/m^2^)	20.5 ± 2.3	20.8 ± 1.7	0.591
**Vital signs**			
Systolic BP (mmHg)	111.2 ± 10.0	106.5 ± 9.3	0.103
Diastolic BP (mmHg)	73.8 ± 7.8	72.5 ± 7.1	0.562
Pulse rate (beats per minute)	74.2 ± 19.5	72.3 ± 8.2	0.663
**Substance use**			
Caffeine (cups per day)	0.6 ± 0.8	0.8 ± 0.6	0.434
Alcohol (cups per day)	0.4 ± 0.4	0.7 ± 0.7	0.100
**Menstrual characteristic**			
Age at menarche (year)	12.9 ± 1.5	13.0 ± 2.6	0.896
Menstrual cycle (days)	29.5 ± 1.6	29.0 ± 1.6	0.299
Duration of menstruation (days)	5.1 ± 1.1	5.8 ± 2.3	0.201
**Dysmenorrhea measures**			
Maximum VAS score before the visit	2.1 ± 1.0	6.4 ± 1.4	0.000
MMP score	2.2 ± 1.2	5.9 ± 1.0	0.000
CMSS score (duration)	9.0 ± 6.4	25.9 ± 10.2	0.000
CMSS score (magnitude)	7.2 ± 4.9	20.6 ± 6.2	0.000
**Pattern identification for PD**			
Stagnation of *qi* and *blood*	—	22 (95.7%)	
Deficiency of *qi* and *blood*	—	1 (4.3%)	

Continuous variables are summarized as the mean and standard deviation, and categorical variables are summarized as the frequency and proportion. P-values were derived from independent two-sample t-tests between healthy subjects and PD patients. BP: blood pressure; VAS: visual analogue scale; MMP: measurement of menstrual pain; CMSS: Cox menstrual symptom scale.

### Comparison of the radial pulse indices between the PD patient and healthy subject groups

**Figure 1:**
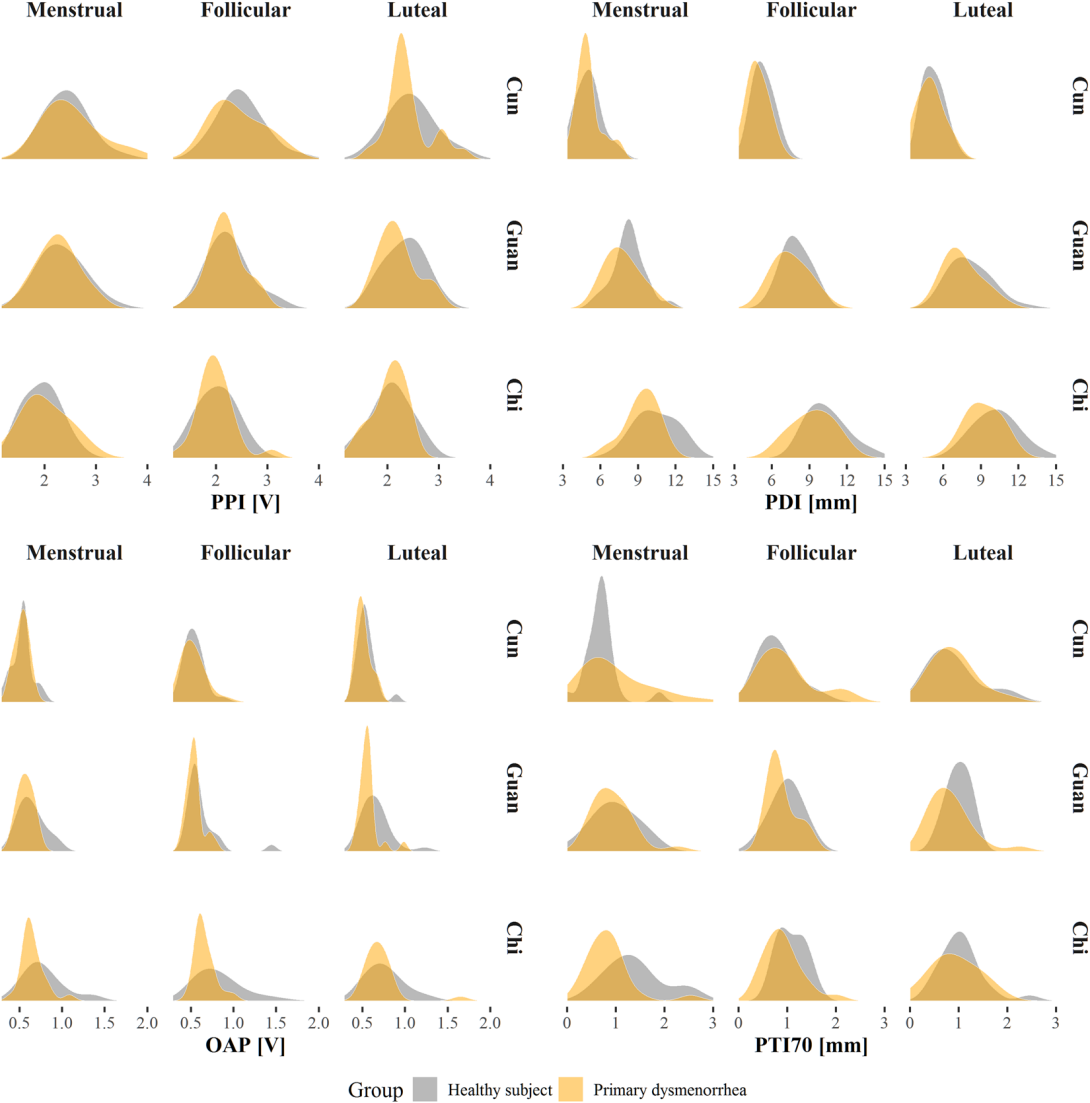
Estimated distributions of the radial pulse indices according to menstrual periods and measured locations.

According to TKM theory, different patterns have different pulse wave characteristics. Therefore, the data for the one patient diagnosed with deficiency of *blood* and *qi* were excluded from the final analysis of radial pulse indices. In total, 46 participants were assigned to the PD patient (n = 22) and healthy subject (n = 24) groups. First, we compared the distributions of radial pulse indices between the patient and healthy subject groups at the *cun*, *guan*, and *chi* (Fig. 1). Since a strong correlation was found between the PTIs, only the PTI70 is provided as a representative parameter, and the distribution data of the PTI20, 30, 50, 80 and 90 are provided in Appendix 1. By comparing distributions, the trends of radial pulse indices between the two groups were found to be similar at the *cun* and *guan*, but the tendency was different between the two groups at the *chi*. Therefore, an independent t-test and multiple logistic regression analysis focusing on the radial pulse indices at the *chi* were performed. Table 2 shows the differences in the PPI, PDI and OAP results between the patients and healthy subjects throughout menstruation after adjusting for age, body mass index, systolic blood pressure, pulse rate, caffeine, and alcohol consumption per day. No significant difference in the PPI was found between the two groups throughout the menstrual cycle. The PDI in the menstrual phase was significantly lower in the PD patient group than that in the healthy subject group (P < 0.01). Similar to the menstrual phase, the PDIs in the follicular and luteal phases were also significantly lower in the PD patient group than those in the healthy subject group (P < 0.05 and P < 0.05, respectively). Although the OAPs in the menstrual and luteal phases were not significantly different, the OAP showed a similar trend in which the marginal means of the OAP in the patient group were lower than those in the healthy subject group (P < 0.10). In contrast, the OAP in the follicular phase was significantly lower in the patient group than that in the healthy subject group (P < 0.01). Table 3 presents the differences in the PTIs (20, 30, 50, 70, 80 and 90) between the PD patient and healthy subject groups throughout the menstrual cycle. The PTIs in the menstrual phase were significantly lower in the PD patient group than those in the healthy subject group (P < 0.05, P < 0.05, P < 0.01, P < 0.001, and P < 0.05, respectively), except for the PTI90 (P < 0.10). However, no significant differences were found in any PTIs between the two groups in the follicular and luteal phases.

**Table 2:** The estimated marginal means of the PPI, PDI, and OAP at each menstrual phase and the differences between the PD patient and healthy subject groups (n = 46).

Variable	Menstrual phase	Healthy subjects (n = 24)	PD patients (n = 22)	Mean difference (95% CI)	Effect size
PPI	Menstrual	1.92 (1.76, 2.08)	2.02 (1.86, 2.18)	0.10 (−0.13, 0.32)	0.24
	Follicular	2.01 (1.85, 2.16)	2.00 (1.84, 2.16)	−0.01 (−0.24, 0.22)	0.03
	Luteal	2.08 (1.92, 2.24)	2.04 (1.88, 2.20)	−0.04 (−0.27, 0.19)	0.10
PDI	**Menstrual**	**10**.**49** (**9**.**92**, **11**.**05)**	**9**.**31** (**8**.**74**, **9**.**89)**	−**1**.17** (−**1**.**99**, −**0**.**35)**	**0**.**83**
	Follicular	10.36 (9.80, 10.92)	9.30 (8.73, 9.87)	−1.06* (−1.88, −0.24)	0.76
	Luteal	10.11 (9.54, 10.68)	9.19 (8.62, 9.76)	−0.92* (−1.74, −0.10)	0.65
OAP	Menstrual	0.76 (0.68, 0.84)	0.66 (0.57, 0.74)	−0.11^^^ (−0.22, 0.01)	0.54
	Follicular	0.82 (0.74, 0.90)	0.66 (0.58, 0.74)	−0.16** (−0.28, −0.05)	0.81
	Luteal	0.76 (0.68, 0.84)	0.72 (0.64, 0.80)	−0.04 (−0.16, 0.07)	0.22

Each value represents the estimated marginal mean and (95% CI) obtained by linear mixed effect models for each variable adjusted for age, BMI, systolic BP, pulse, and caffeine and alcohol consumption per day. ***P < 0.001; **P < 0.01; *P < 0.05; ^P < 0.10.

**Table 3:** The estimated marginal means and group differences for the PTIs at each menstrual phase (n = 46).

Variable	Menstrual phase	Healthy subjects (n = 24)	PD patients (n = 22)	Mean difference (95% CI)	Effect size
PTI20	**Menstrual**	**3**.**20** (**2**.**86**, **3**.**53)**	**2**.**59** (**2**.**25**, **2**.**94)**	−**0**.60* (−**1**.**09**, −**0**.**12)**	**0**.**72**
	Follicular	2.84 (2.51, 3.17)	2.71 (2.38, 3.05)	−0.12 (−0.61, 0.36)	0.15
	Luteal	2.81 (2.47, 3.15)	2.64 (2.30, 2.98)	−0.17 (−0.65, 0.32)	0.20
PTI30	**Menstrual**	**2**.**74** (**2**.**42**, **3**.**06)**	**2**.**18** (**1**.**86**, **2**.**51)**	−**0**.56* (−**1**.**02**, −**0**.**09)**	**0**.**69**
	Follicular	2.44 (2.13, 2.76)	2.22 (1.90, 2.55)	−0.22 (−0.68, 0.24)	0.27
	Luteal	2.31 (1.98, 2.63)	2.19 (1.87, 2.51)	−0.12 (−0.58, 0.34)	0.15
PTI50	**Menstrual**	**1**.**90** (**1**.**66**, **2**.**14)**	**1**.**34** (**1**.**10**, **1**.**59)**	−**0**.56** (−**0**.**90**, −**0**.**21)**	**0**.**92**
	Follicular	1.60 (1.37, 1.84)	1.47 (1.22, 1.71)	−0.14 (−0.49, 0.21)	0.23
	Luteal	1.59 (1.35, 1.84)	1.43 (1.18, 1.67)	−0.17 (−0.51, 0.18)	0.28
PTI70	**Menstrual**	**1**.**41** (**1**.**22**, **1**.**60)**	**0**.**87** (**0**.**68**, **1**.**06)**	−**0**.54*** (−**0**.**81**, −**0**.**27)**	**1**.**15**
	Follicular	1.08 (0.89, 1.26)	0.92 (0.74, 1.11)	−0.15 (−0.42, 0.11)	0.33
	Luteal	1.01 (0.82, 1.19)	0.92 (0.74, 1.11)	−0.09 (−0.35, 0.18)	0.18
PTI80	**Menstrual**	**0**.**99** (**0**.**82**, **1**.**16)**	**0**.**72** (**0**.**54**, **0**.**89)**	−**0**.27* (−**0**.**52**, −**0**.**03)**	**0**.**64**
	Follicular	0.68 (0.51, 0.85)	0.62 (0.45, 0.80)	−0.06 (−0.30, 0.19)	0.14
	Luteal	0.75 (0.58, 0.92)	0.69 (0.52, 0.87)	−0.06 (−0.30, 0.19)	0.14
PTI90	**Menstrual**	**0**.**59** (**0**.**46**, **0**.**71)**	**0**.**42** (**0**.**29**, **0**.**55)**	−**0**.**17**^^^ (−**0**.**35**, **0**.**01)**	**0**.**53**
	Follicular	0.39 (0.27, 0.52)	0.36 (0.24, 0.49)	−0.03 (−0.21, 0.15)	0.09
	Luteal	0.47 (0.34, 0.60)	0.31 (0.19, 0.44)	−0.16^^^ (−0.34, 0.02)	0.51

All details in the table are identical to those in Table 1. ***P < 0.001; **P < 0.01; *P < 0.05; ^P < 0.10.

### Associations between primary dysmenorrhea and radial pulse indices

**Figure 2:**
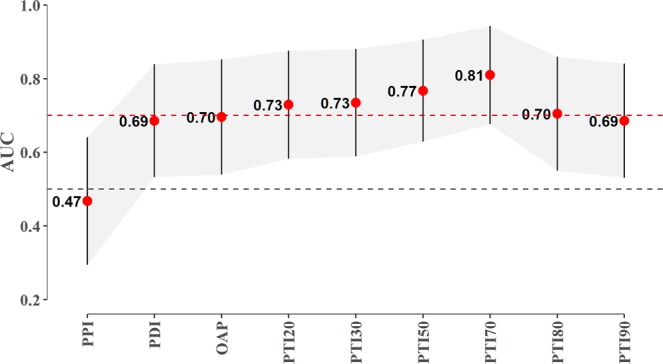
The area under the ROC curve for each radial artery pulse index for PD status based on the univariate logistic regression results. Error bars for each AUC value within the shaded region indicate the 95% confidence interval for the AUC of each index, which was derived from DeLong’s method. The black and red dashed lines indicate the AUC values of 0.5 and 0.7, respectively.

**Figure 3:**
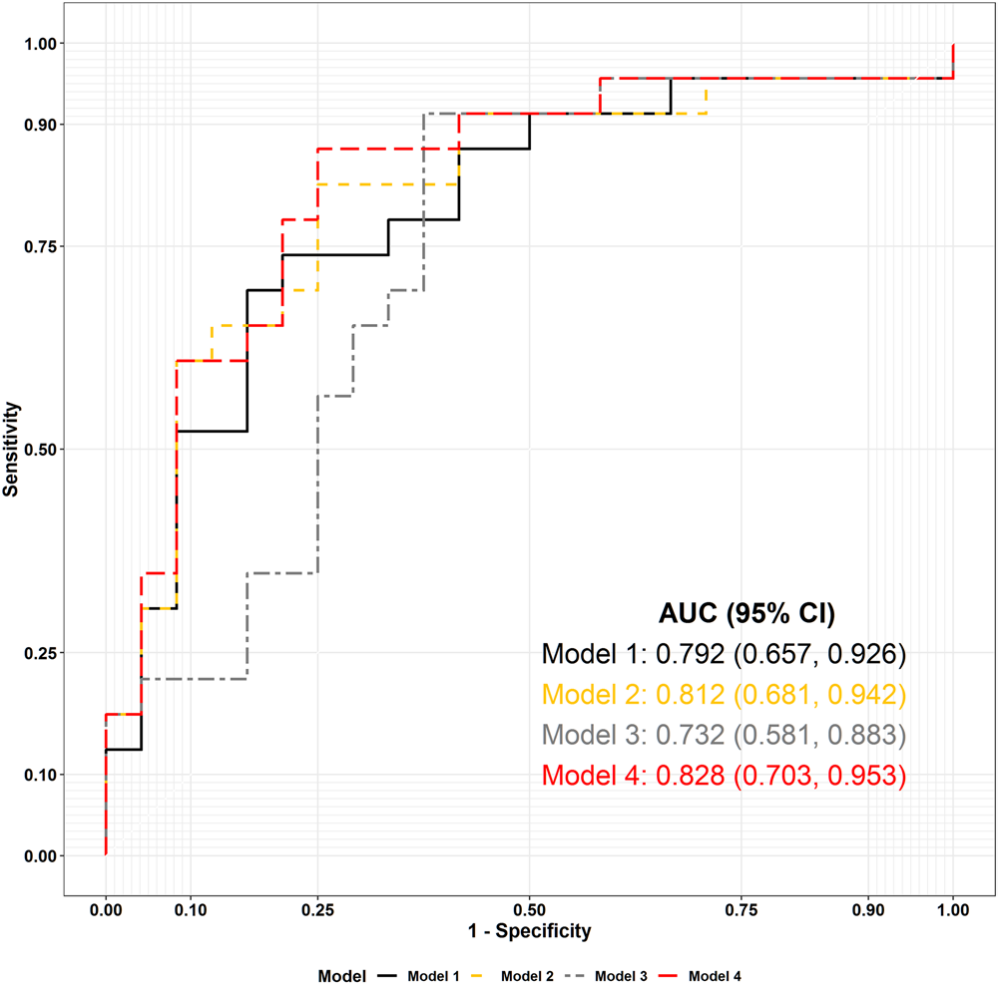
ROC curves of the predicted probabilities for each multiple logistic regression model based on leave-one-out cross validation. The 95% CIs of the AUCs for each model were estimated using DeLong’s method.

We calculated the area under the ROC curve to investigate the relationship between PD and each radial artery pulse variable measured at the *chi* in the menstrual phase. The areas under the ROC curves of the radial pulse indices for PD status were drawn based on the univariate logistic regression results. In Fig. 2, the error bars for each AUC value within the shaded region indicate the 95% confidence interval for the AUC of each index, which was derived from DeLong’s method. The black and red dashed lines indicate AUC values of 0.5 and 0.7, respectively. The PTI70 was found to be the best predictive index for the radial pulse (AUC = 0.81) among all PTI indices. The AUC value for the OAP was 0.70, whereas the AUC value for the PDI was 0.69. The PPI had the lowest AUC value of all indices (AUC = 0.47), as shown in Fig. 2. Table 4 presents the multiple logistic regression results for the associations of the radial pulse indices with the likelihood of a menstrual severity score. We calculated the AIC to estimate the quality of each model compared to that of each of the other models by assessing the relative information loss in a given model, where less information loss corresponds to higher model quality. The results of our modeling showed that Model 4 had the lowest AIC and the highest P-value among the models (AIC = 50.193, P = 0.590), indicating that Model 4 had the highest quality compared to the other models. The performance of Model 4 was assessed by calculating the area under the ROC curve, which was 0.828 (95% CI 0.703 to 0.953), reflecting nearly perfect discrimination (Fig. 3).

**Table 4:** Likelihood ratio tests for the multiple logistic regression results for PD status using different radial pulse indices.

Model	Description	−2LL	AIC	df	Δ-2LL	Test	P-value
Model 1	y ~ PPI + PDI + OAP + PTI70	43.137	53.137	42			
Model 2	y ~ PDI + OAP + PTI70	43.476	51.476	43	−0.340	1 vs. 2	0.560
Model 3	y ~ PDI + PTI70	51.361	57.361	44	−8.225	1 vs. 3	0.016
**Model 4**	**y ~ OAP + PTI70**	**44**.**193**	**50**.**193**	**44**	**−1**.**057**	**1 vs**. **4**	**0**.**590**

## Discussion

PD is defined as cramp-like pain in the lower abdomen before or during menstruation without any related diseases^13^. TKM holds that PD is often caused by *blood* stasis blocking the uterus; that is, during menstruation, *qi* and *blood* fail to freely flow^19,25^. Apart from pain, changes in the movements of *qi* and *blood* also affect the radial pulse. In this study, the pulse waves of all participants were collected during the follicular, luteal and menstrual phases using a PTD. Then, radial pulse indices measured from PD patients and healthy subjects were compared.

This study began with the pattern identification and confirm the pattern of PD patients. As a result, 22 of 23 patients were diagnosed with stagnation of *qi* and *blood*. Stagnation of *qi* and *blood* has been considered a key etiological factor in the pathology of PD^19^. The stagnation of *qi* and *blood* leads to obstruction of the menstrual flow in the uterus, which causes pain^25^. The most commonly reported pattern identified among PD patients has been stagnation of *qi* and *blood*^20^. Therefore, the large number of PD patients diagnosed with stagnation of *qi* and *blood* pattern in this study was considered equitable.

In this study, we focused our analysis only on the *chi* pulse as the distributions of *cun* and *guan* pulses were similar between the two groups. The methods of pattern differentiation in gynecology are generally based on viscera pattern differentiation. The *Kidney* stores essences and dominates reproduction. If *Kidney* is deficient, then instability of the thoroughfare vessel (the *chong* meridian) and conception vessel (the *ren* meridian) can occur, which can induce many gynecology disorders. In addition, the *chi* pulse on the left hand is associated with *Kidney yin*, while *chi* pulses on the right hand correspond to *Kidney yang* and the uterus. The affiliation between *Kidney* and gynecology may explain why only the *chi* pulse showed differences in the distribution tendency of our result^26^.

A comparison of the pulse indices between the PD patients and healthy subjects throughout menstruation showed that significant differences were identified between the two groups during the menstrual phase but not during the follicular or luteal phases. Among the pulse indices, PDI and PTI showed significant differences for the two groups during the menstrual phase. Although OAP did not show significant differences between the two groups during the menstrual phase, OAP showed a similar tendency. These results may be due to the hormonal changes during the menstrual period when prostaglandin is excreted. The prostaglandins, in particular, cause intense vasoconstriction and affect the vessel wall tension, which eventually leads to changes in the pulse wave^27^. Therefore, the pulse characteristics before menstruation begins, at the start of menstruation, and the day after menstruation begins may be different according to the excretion of prostaglandin. According to TKM and TCM textbooks, patients with typical stagnation of *qi* and *blood* generally have a string-like pulse, which is also known as a wiry pulse, whereas the deficiency of *blood* and *qi* pattern can be manifested as a deep and slow pulse^19^.

Together with the above statements, the pulse signals measured by the PTD in this study could be considered to have characteristics of a string-like pulse. A string-like pulse can be described as a straight, long and taut pulse, which is similar to a musical string when touched. According to a modern expression with quantifiable physical properties, the major property of string-like pulses is tension, and depth and strength properties can also affect this type of pulse.

The PTI developed in this study was inspired by the Delphi research conducted in Kyung Hee University Korean Medicine Hospital (KHUKMH)^28^. This research was performed to study the descriptions of physical properties of specific pulses based on a consensus among experts and medical literature. The experts involved in this study agreed on a description of pulse tension as a section for which the degree of changes in increasing pulse amplitude according to the applied pressure is below a certain level. However, a pulse tension algorithm using a P-H curve has not yet been reported. A P-H curve showing the pulse pressure as a function of the applied pressure was originally used to determine whether the pulse was strong when the applied pressure was weak or strong^1^. In other words, this applied pressure-based method is considered more suitable for estimating pulse depth rather than pulse tension.

Instead, a sensor displacement-based method was used to calculate the PTI because the CETM was applied to the PTD in this study. The pulse pressure as a function of actual sensor displacement was obtained, and the PTI was calculated. The overall increasing tendency of the pulse pressure according to the applied pressure can be reflected by the PTI. Since this was the first study on a quantitative index for pulse tension, the various PTI values corresponding to the 20, 30, 50, 70, 80, and 90 percentages of the maximum pulse amplitude were first calculated, and then the index that best reflected pulse tension was examined. In this study, the PTI70 had the largest effect size and AUC value; therefore, multiple logistic regression models with the PTI70 were constructed. For the PTI80 and PTI90, since the time interval between the maximum pulse amplitude point and the point greater than 80% or 90% of the maximum pulse amplitude was very short, the accuracy of the increasing trend for pulse pressure was limited. Furthermore, in addition to the PTI, the OAP was also considered to estimate pulse tension. Because a pulse with high tension exerts a strong resistive force against applied pressure, the pulse amplitude increases rapidly, even when only light pressure is applied. Therefore, a low OAP may reflect high pulse tension as well as low pulse depth. The OAP value of the PD patients was lower than that of the healthy subjects, as expected.

Pulse depth is another property of a string-like pulse. Although both string-like and tight pulses have identical characteristics, including a high degree of tension, a shallow depth is a typical characteristic of a string-like pulse, whereas a deep depth is a characteristic of a tight pulse, although this characterization is controversial. In this study, the PDI of the PD patients was significantly lower than that of the healthy subjects. The PD patients were considered to have a string-like pulse, which is consistent with TKM theory, in which stagnation of *qi* and *blood* corresponds to a string-like pulse. Consequently, throughout the PDI, OAP and PTI, it can be concluded that the radial pulse of the patients with PD appeared string-like, whereas the radial pulse of the healthy subjects appeared normal.

In our study, we performed multiple logistic regression for the associations of radial pulse indices with the likelihood of a given menstrual severity score using four indices, including the PPI, PDI, OAP, and PTI70. Model 4 had the highest quality among the tested models. Moreover, the area under the ROC curve was 0.828, which also showed that this model was an excellent classifier. Only the PTI70 and OAP, which are related to pulse tension, were selected for Model 4 among the four indices since tension indices were considered to be potentially more relevant to pain than to other characteristics. This finding is consistent with reports from previous studies that pain is often associated with a string-like pulse, whose main property is pulse tension^18^. Our results imply that pulse tension is an important major pulse characteristic of patients with PD and suggest that the PTI and OAP may be potential clinical indicators for pain disorders.

This study has two limitations. First, this study was designed as a single-center, prospective case-control study without randomization, blinding or allocation concealment. Therefore, the results of this study should be interpreted with caution. Second, this study could not explore the differences in the radial pulse indices according to pattern identification because all subjects were diagnosed with stagnation of *qi* and *blood*, with the exception of only one subject who was diagnosed with a deficiency in *qi* and *blood*. Third, this study could not explore the differences among pulse characteristics according to various types of pain, although tense pulse was expected as a major pulse characteristic. There was no previous study about a pulse difference among different types of pain. Because potential clinical indicators for menstrual pain were investigated in this study, further study for other types of pain based on radial pulse indices such as PTI and OAP will be of great value. We will conduct a large, multicenter trial that considers various pattern identification and types of pain after further reviewing the results of this study.

Despite these limitations, we believe that this study provides a basis for further and more comprehensive studies. This study is the first among the current literature to investigate radial pulse indices focusing only on the *chi* pulses of PD patients throughout the menstrual cycle using a PTD. In addition, pattern identification for the PD patients was observed, ensuring more accurate and reliable results. Furthermore, this is the first study to analyze pulse tension by developing the PTI and applying it to clinical practice. This information on pulse depth, width, length, regularity, and tension can be used as a reference for medical treatment guidelines in acupuncture, moxibustion, and herbal medicine. Hence, the objective and quantitative pulse diagnosis is one of the most important clinical processes. The results of this study confirmed the possibility of quantitatively measuring pulse tension, which could also provide a scientific perspective on the development of the PTD.

## Materials and Methods

### Study design

This prospective, case-control clinical study was conducted at the KHUKMH in Seoul, Republic of Korea. The experimental protocol was approved by the Ethics Committee of the Institutional Review Board in KHUKMH, and the approval number was KOMCIRB-150622-HR-021. This trial was registered with the Korean Clinical Trials Registry (identifier number KCT0001604) and was conducted in accordance with the principles of the Declaration of Helsinki.

### Participants

The subjects were enlisted through recruitment posters displayed around Kyung Hee University and KHUKMH. All subjects were required to visit KHUKMH and were asked to sign the informed consent forms after receiving a full explanation of the study. This study enrolled 48 women aged from 20 to 29 years who were subsequently stratified according to inclusion and exclusion criteria. Furthermore, all subjects were obligated to provide their sociodemographic data and completed measurements of menstrual pain (MMP) and a Cox menstrual symptom scale (CMSS) questionnaire^29,30^.

### Inclusion and exclusion criteria

The inclusion criteria for the participants were predefined as follows: (1) females between 20 and 30 years of age; (2) a normal menstrual cycle (lasting 28 ± 3 days) for the past 3 months; (3) the ability to communicate with the clinical researchers; (4) the ability to voluntarily agree to participate in this clinical study; and (5) the ability to provide written informed consent. The exclusion criteria were as follows: (1) secondary dysmenorrhea caused by uterine myoma, endometriosis or infection of the genitals confirmed by pelvic examination, an ultrasound, and laboratory tests; (2) a history of medical operations or procedures; (3) a history of major neuropsychiatric disorder or antidepressant, anti-serotonin agent, barbiturate, or psychotropic drug use within the past 3 months; (4) a history of major medical diseases, including hypertension, diabetes, hyperlipidemia, gastritis, enteritis, gastroesophageal reflux disease, and Crohn’s disease; (5) extreme dieting within one week prior to study initiation; (6) pregnancy determined by a urine pregnancy test, planned pregnancy or lactation; (7) congenital vascular anomalies or a history of wrist fracture; (8) participation in another clinical study in the previous month; and (9) exclusion at the investigator’s discretion.

### Sample size

Calculation of the required sample size was guided by a previous study^31^. In this previous study, the systolic blood pressure showed a significant difference between the healthy subject group and PD patient group at the luteal phase. The systolic blood pressure is closely related to the pulse pressure^32^, and the pulse pressure was considered as the primary outcome at the time of this clinical study design. Therefore, the effect size of the pulse pressure was estimated as 0.83, which was obtained from the result of the systolic blood pressure described in Singh *et al*. With a statistical significance level of 0.05 (2-sided test) and a study power of 80%, the estimated sample size was calculated to be 19 participants per group (patient and healthy subject groups). With consideration of a 20% of drop-out rate, 24 subjects were recruited for each group. Consequently, the total sample size was 48 subjects with a 1:1 ratio for group allocation.

### Classification of the patterns in PD patients

Based on the TKM pattern identification protocol for women with PD, three TKM physicians with ten years of clinical experience independently diagnosed the PD patients’ patterns (stagnation of *qi* and *blood*, coagulation of cold-dampness, deficiency of *qi* and *blood*, or impairment of the liver and kidneys)^20^. All three physicians were blinded to the diagnostic results. Participants with confusing patterns or an inconsistent diagnosis among the physicians were excluded from the analysis.

### Experimental procedures

A research investigator surveyed the demographic characteristics. After a screening test was completed, the allocation process was performed, and the 48 enrolled subjects were divided into two groups according to visual analogue scale scores (VAS score ≥ 4 for the PD patient group and VAS score < 4 for the healthy group)^31^. The VAS is commonly used to measure menstrual pain intensity and consists of a 10-cm horizontal line with “no pain” on one end and “worst possible pain” on the other end. All subjects were asked to rate their pain intensity to evaluate menstrual pain.

After completing the allocation process, the research investigator calculated the assessment period of each subject by considering her menstrual cycle length for the previous 3 months. Upon completion of the run-in period, the assessments were conducted at the beginning of menstruation, the follicular phase (the ninth day of the menstrual cycle) and the luteal phase (the 22nd day from the start of menstruation). Another licensed physician who is an assessor evaluated the outcome variables and recorded vital signs as well as any adverse events during the assessment period. Any additional relevant information was provided by the subjects.

### The pulse tonometric device

Pulse waves were obtained using a PTD (KIOM-PAS ver.2.0, Korean Institute of Oriental Medicine, Republic of Korea). The electromechanical stability of the device was certified based on the IEC 60601-1 2^nd^ edition, and the reliability and safety of the device were confirmed based on clinical good manufacturing practice in Korea^33^. The PTD consists of a main body with an arm holder and a sensing body attached to a mobile actuator. The six-channel piezoresistive sensor, arrayed in a row attached to the end of the actuator where the sensing element has dimensions of 1 × 1 square millimeters, was used to measure the pulse signal. The sensor array was positioned in a row at each palpation position. The *guan* is positioned at the center of the radial styloid at the wrist, where the tip of the physician’s middle finger is placed; the *cun* is positioned next to the *guan* on the distal side, where the tip of the physician’s index finger rests; and the *chi* is positioned on the proximal side, where the tip of the physician’s ring finger is placed. The responsive pulse signal was acquired at a sampling rate of 1000 Hz, and the continuously evolving tonometric mechanism (CETM) was applied to the PTD^2^. The CETM is described as follows: After positioning the sensor at three palpation positions, the sensor was gradually moved downward at 0.09 mm/sec until reaching the optimal applied pressure (OAP), where the pulse amplitude reached its maximum. Then, the sensor was stopped to maintain the applied pressure for 70 seconds. A trained operator measured the pulse signals according to standard operating procedures. After the removal of noise and the baseline wander of the acquired pulse signal, feature points were extracted.

### Pulse analysis indices

**Figure 4:**
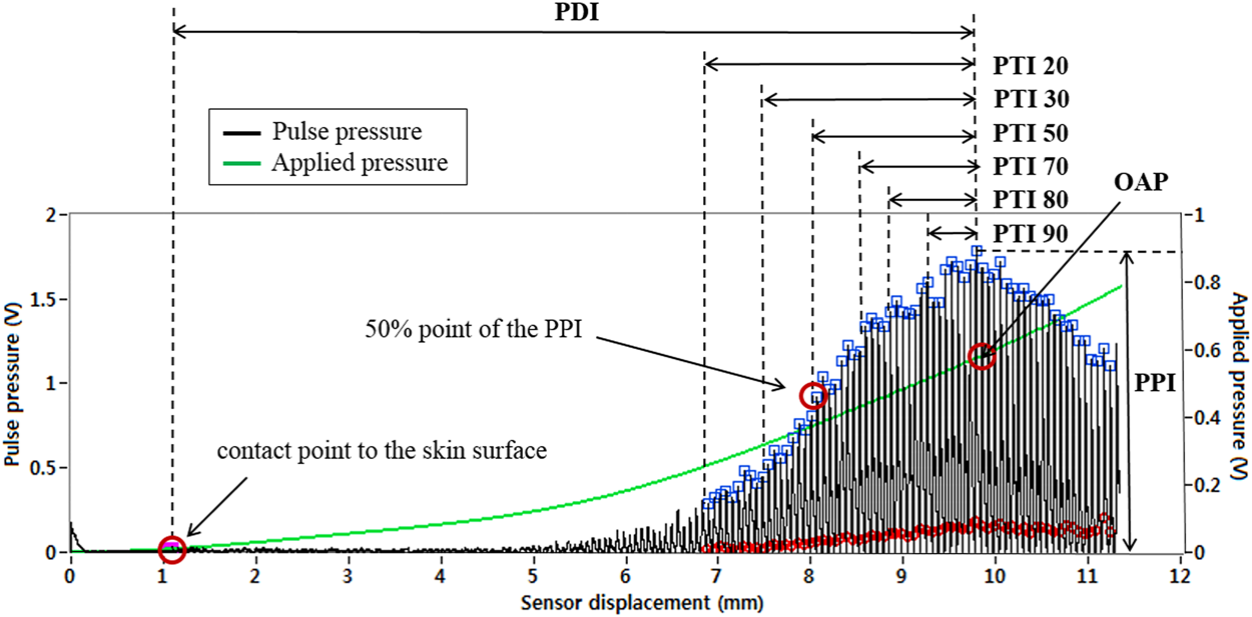
The pulse pressure and applied pressure signal with the analysis indices.

Pulse tension is a major pulse characteristic of patients with pain such as menstrual pain, headaches and chest pain^18^. Therefore, a quantitative index reflecting pulse tension is critical to compare pulse signal characteristics between PD patients and healthy subjects. As the pressure applied to the pulse increases, the responsive pulse pressure generally increases until the blood vessel starts to deflate, which can be observed on the pulse signal measured by the CETM. A pulse with a high degree of tension exhibits strong resistive force against the applied pressure. Therefore, the pulse amplitude rapidly increases according to the applied pressure, and the maximum pulse amplitude is quickly reached. However, since a pulse with low tension exhibits strong compliance against the applied pressure, the pulse amplitude slowly increases as the applied pressure increases; therefore, the maximum pulse amplitude is slowly reached. To quantify the force of these resistive pulse sections against the applied pressure, the pulse tension index (PTI) was developed. Each time interval between the maximum pulse amplitude point and the starting points with greater than 20%, 30%, 50%, 70%, 80%, and 90% of maximum pulse amplitude was calculated from the pulse signal measured by the CETM. Subsequently, the six indices were converted to motor displacement units of the PTD. Finally, the overall increasing tendency of the pulse pressure according to the applied pressure could be observed by calculating the PTI. Lower PTIs may reflect the entire increasing trend, including the initial sections, whereas higher PTIs may indicate a later increasing trend. Since no studies on a quantitative index for pulse tension are available, all six indices were first analyzed, and the index that best reflected the pulse tension was examined. In addition, the applied pressure value may be different even though the pulse has the same PTI value. Therefore, the OAP, one of the basic indices for pulse characteristics, was also calculated to estimate pulse tension characteristics. Pulse depth is one of the most important pulse characteristics. Pulse depth was measured based on the displacement of the sensor in the direction normal to the skin surface, and the pulse depth index (PDI) was calculated^33^. In addition, strength properties can also affect pulses. The voltage response of the sensor when the maximum pulse amplitude was reached was calculated as the pulse pressure index (PPI) to estimate the basic pulse strength. Table 5 shows the definitions and descriptions of the analysis indices in this study, and Fig. 4 illustrates the pulse pressure and applied pressure signal with the analysis indices.

**Table 5:** Analysis indices used in this study.

Index	Definition (unit)	Description
PPI	Pulse pressure index (V)	Voltage response upon reaching the maximum pulse amplitude, which is the largest value between the starting point and peak point of each pulse signal
PDI	Pulse depth index (mm)	Sensor displacement from the point of contact with the skin surface to the maximum pulse amplitude point
PTIx	Pulse tension index (mm)	Sensor displacement between the maximum pulse amplitude point and the starting points with greater than x percentage of the maximum pulse amplitude (x = 20, 30, 50, 70, 80, 90)
OAP	Optimal applied pressure (V)	The applied pressure at which the pulse amplitude reaches its maximum

### Statistical analysis

All statistical analyses were performed using R version 3.5.1^2^. The significance level for all statistical tests was set to α = 0.05. The demographic and baseline characteristics of the participants in this study were summarized as the mean and standard deviation (SD). Independent two-sample t-tests were applied to compare the mean differences in baseline values between the patient and healthy subject groups. Linear mixed effects (LME) models for each radial artery pulse parameter in the *chi* location were established to estimate the marginal means of both the patient and healthy subject groups for each stage of the menstrual cycle. Several confounders, such as age, BMI, systolic BP, pulse, and the amounts of caffeine and alcohol consumption (cups/day), were considered covariates in the LME models. The mean differences between the patient and healthy subject groups at each period of the menstrual cycle were tested based on the estimated marginal means from the LME models. The effect sizes in Tables 2 and 3 were calculated using the equation suggested by Rosnow *et al*.^34^. To investigate the associations between radial artery pulse markers and PD, the area under the receiver operating curve (ROC) for each radial artery pulse parameter was obtained. Multiple binary logistic regressions with different combinations of pulse parameters based on four distinct characteristics were applied to determine the best fitting model. The likelihood ratio test and Akaike information criterion (AIC) were employed to evaluate model performance^35^. For further analysis, ROC curves and their AUCs for each model were obtained based on leave-one-out cross validation.

## Supplementary information

Appendix 1
