# Supplementary material for: A comparative study of the radial pulse between primary dysmenorrhea patients and healthy subjects during the menstrual phase
Source: Sci Rep. 2019 Jul 4;9:9716. doi: 10.1038/s41598-019-46066-2 (PMC6609770; doi:10.1038/s41598-019-46066-2)
Supplement: Supplementary file 1 — Appendix 1 [file 41598_2019_46066_MOESM1_ESM.pdf]

A comparative study of the radial pulse between primary dysmenorrhea patients and healthy subjects during the menstrual phase

Jihye Kim[1#], Jang-Han Bae[1#], Boncho Ku[1], Mi Hong Yim[1], Lin Ang[1], Hyun Ho Kim[2], Young Ju Jeon[1*]

[1] Future Medicine Division, Korea Institute of Oriental Medicine, 1672 Yuseongdae-ro, Yuseong-gu, Daejeon, Republic of Korea. [2] Dept. of Acupuncture & Moxibustion Medicine, Dongshin Korean Medicine Hospital (Mokdong, Seoul), 351, Omok-ro, Yangcheon-gu, Seoul, Republic of Korea. Correspondence and requests for materials should be addressed to Y.J.J. (email: jyj92@kiom.re.kr)

[#] These authors contributed equally to this work.

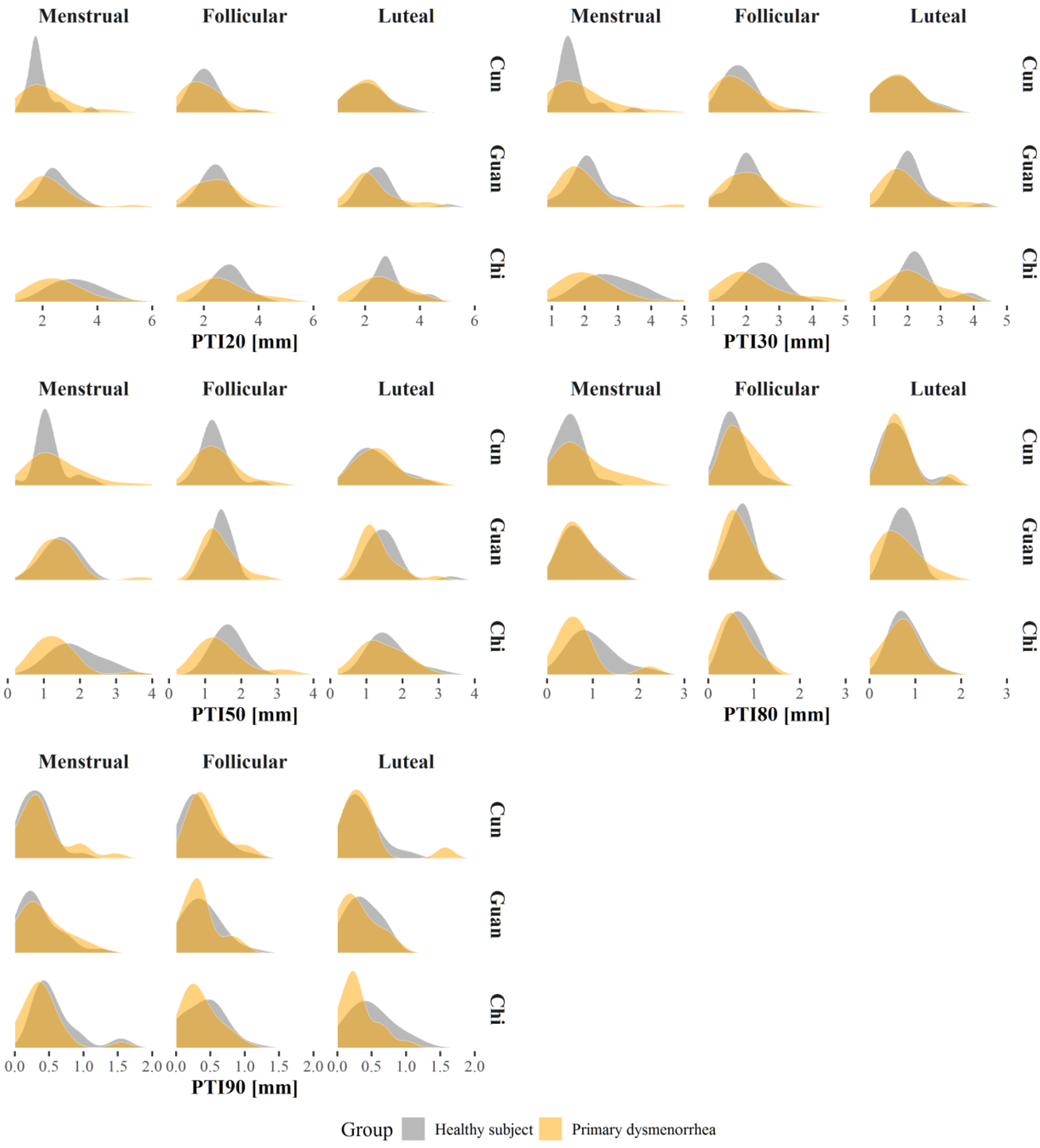

Menstrual
Follicular
Luteal
Cun
Guan
Chi
PTI20 [mm]
PTI30 [mm]
PTI50 [mm]
PTI80 [mm]
PTI90 [mm]
Group
Healthy subject
Primary dysmenorrhea
